# Comparative Study of Hematological Parameters and Biomarkers of Immunity and Inflammation in Patients with Psoriasis and Atopic Dermatitis

**DOI:** 10.3390/medicina59091622

**Published:** 2023-09-08

**Authors:** Mirjana Bakic, Aleksandra Klisic, Vesna Karanikolic

**Affiliations:** 1 Clinic for Dermatovenerology, Clinical Center of Montenegro, 81000 Podgorica, Montenegro; mirjana.bakic@gmail.com; 2 Faculty of Medicine, University of Montenegro, 81000 Podgorica, Montenegro; 3 Center for Laboratory Diagnostics, Primary Health Care Center, 81000 Podgorica, Montenegro; 4 Clinic for Skin Diseases of the Clinical Center Nis, School of Medicine, University of Nis, 18000 Nis, Serbia

**Keywords:** atopic dermatitis, inflammation, psoriasis

## Abstract

*Background and Objectives*: There are no studies regarding comparative analysis of hematological parameters in patients with psoriasis (PsO) and atopic dermatitis (AD), whereas studies examining serum biomarkers of immunity and inflammation in these entities are scarce and contradictory. We aimed to compare such parameters in patients with PsO and AD. *Materials and Methods*: Patients with PsO (*n* = 40) and AD (*n* = 40) were consecutively included in this cross-sectional study. Hematological parameters and biomarkers of immunity and inflammation (interferon-gamma (IFN-γ), interleukine (IL)-22 and C-reactive protein (CRP)) were determined. *Results*: While the mean corpuscular volume (MCV) was higher in the PsO group vs. the AD group (*p* < 0.05), there was no difference in the other examined parameters between groups. A higher neutrophil-to-lymphocyte ratio (NLR) was found in patients with AD > 50 years vs. patients with PsO of similar age (*p* < 0.05). Higher IL-22 levels were found in patients with AD < 50 years vs. patients with PsO of similar age (*p* < 0.05). Lower IL-22 levels were found in patients with AD > 50 years vs. patients with AD < 50 years (*p* < 0.05). Patients with PsO and with comorbidities had lower platelets (PLT), plateletcrit (PCT) and platelet-to-lymphocyte ratio (PLR), whereas lymphocytes, red cell distribution width-to-PLT ratio (RPR) and mean platelet volume/PLT ratio (MPR) were higher vs. PsO patients without comorbidities. Patients with AD and with comorbidities had lower PCT and PLR, whereas RPR was higher vs. AD patients without comorbidities. *Conclusions*: A higher pro-inflammatory state (i.e., higher NLR and IL-22) was found in AD vs. PsO in age-specific groups. A higher pro-inflammatory state (i.e., as reflected by platelet indexes) was found in both diseases with comorbidities.

## 1. Introduction

Chronic skin inflammatory diseases, such as atopic dermatitis (AD) and psoriasis (PsO) are related to the imbalance between anti-inflammatory and pro-inflammatory mediators in favor of the latter ones [1]. Despite their chronic and pro-inflammatory nature, these diseases have some differences in their pathogenesis.

The abnormal T helper 2 (Th2) lymphocyte activation with consequent secretion of a variety of pro-inflammatory cytokines, such as interleukin (IL)-4, IL-5, IL-22, etc., that further leads to the destruction of epidermal barrier is suggested as the underlying pathological mechanism of AD [1]. On the other hand, the activation of abnormal Th1 and Th17 lymphocytes with concomitant secretion of interferon-gamma (IFN-γ), tumor necrosis factor-alpha (TNF-α), IL-17, IL-23, etc. is assumed to be the pathological mechanism of PsO [1].

A variety of these cytokines favor the prothrombotic state followed by platelet hyperactivity [2]. Despite the well-established role of platelets (PLT) in hemostasis, their contribution to the immuno-inflammatory regulatory processes have only recently been described [3,4].

We have previously showed an increase in several hematological markers, such as total white blood cell (WBC) count, PLT count and their indexes in relation to some other chronic disorders, in both youth and adults [5,6,7].

Neutrophils, as the most numerous WBCs, are the components of the innate immune system [8]. The enhanced cytotoxicity of neutrophils is due to their ability to force monocyte recruitment, as well as to activate macrophages that secrete the precursors of interleukins, modulate immune response and precede tissue damage [5,8].

Biomarkers of immunity and inflammation, as well as hematological biomarkers have been studied in both disease entities and separately in comparison with healthy individuals [8,9,10,11]. However, to the best of our knowledge, there are no studies regarding comparative analysis of hematological parameters in patients with AD and PsO, whereas studies examining serum biomarkers of immunity and inflammation in these entities are scarce and contradictory. Hence, we aimed to compare such parameters in patients with PsO and AD to get a deeper understanding of the pathophysiological traits of these disease entities.

## 2. Materials and Methods

### 2.1. Patients

The current cross-sectional study recruited a total of 40 patients with AD and 40 patients with PsO. Once the Institutional Ethics Committee approved the study protocol, the research was carried out in accordance with the ethical principles of Helsinki Medical Declaration. The written informed consent was signed by all participants.

The questionnaire (consisting of lifestyle habits, demographic data, comorbidities and medication use) was given to each patient to be filled it in. The inclusion criteria were patients diagnosed with AD or PsO who were not receiving any kind of biologic therapy. Patients with skin diseases other than AD or PsO, malignant diseases, stroke, mental disorders, pregnant women and those with C-reactive protein (CRP) ≥ 10 mg/L were excluded from the study.

### 2.2. Methods

The venipuncture was conducted in the morning after an overnight fasting state. The whole blood samples were taken in K2EDTA tubes for the determination of hematological parameters, whereas the other samples were taken in serum separator and clot activator tubes for the measurement of CRP, IFN-γ and IL-22.

The hematological parameters were determined on a Sysmex XN-1000 analyzer (Sysmex Corporation, Kobe, Japan) automatically, as a part of a complete blood cell count.

The indexes were calculated [5,6,7] as following: NLR = neutrophil-to-lymphocyte ratio, PLR = platelets (PLT)/lymphocytes ratio, RPR = red cell distribution width (RDW)/PLT ratio and MPR = mean platelet volume (MPV)/PLT ratio.

Serum CRP levels were determined on a Roche Cobas c501 chemistry analyzer (Roche Diagnostics GmbH, Mannheim, Germany), whereas IFN-γ and IL-22 were measured by an enzyme-linked immunosorbent commercial assay (ELISA).

### 2.3. Statistical Analysis

Statistical analysis was performed using the SPSS statistical package (version 18.0 for Windows, SPSS, Chicago, IL, USA). The Shapiro–Wilk test was applied for testing the distribution of variables.

Data are presented as median (interquartile range) for continuous variables or as counts and percentages for categorical variables. The differences between groups were evaluated by Mann–Whitney U test or Kruskal–Wallis test for continuous data or by chi-square test for categorical data. *p* level < 0.05 was considered to be statistically significant.

## 3. Results

The current study included an equal total number of patients with PsO and AD (*n* = 40, i.e., 18 women with PsO, 22 men with PsO, 22 women with AD and 18 men with AD) with an insignificant difference in gender distribution between the groups (*p* > 0.05). The median (IQ) age in patients with PsO was 50 (39–67) years, whereas the median age in patients with AD was 42 (34–51) (*p* < 0.05).

Nearly half of the patients in both groups had comorbidities (*n* = 19 in the PsO group vs. *n* = 20 in the AD group) (*p* = 0.129), with hypertension, type 2 diabetes mellitus and asthma as the most prevalent ones. A total of 35% patients in both groups had hypertension (*n* = 14 in both groups), 20% (*n* = 8) of patients with PsO had type 2 diabetes mellitus compared with 10% (*n* = 4) of patients with diabetes in the AD group and 17.5% (*n* = 7) of patients had asthma in the AD group compared to 5% (*n* = 2) patients with asthma in the PsO group.

Examined hematological parameters in patients with PsO vs. AD are presented in Table 1.

Except for MCV, which was significantly higher in PsO patients compared with patients with AD (*p* = 0.040), there was no difference in hematological parameters between patients with PsO and AD. In comparison with patients with PsO, MCH was lower and RDW was higher in patients with AD, but with borderline statistical significance (*p* = 0.064 and *p* = 0.058, respectively).

There was no difference in biomarkers of immunity and inflammation in patients with PsO vs. AD. NLR was higher in patients with AD, but with borderline statistical significance (*p* = 0.100, Table 2).

Since patients with PsO were generally older than patients with AD, we performed further statistical analysis between the PsO and AD subgroups according to age, i.e., younger or older than 50 years, and only found differences in NLR and IL-22.

**Figure 1:**
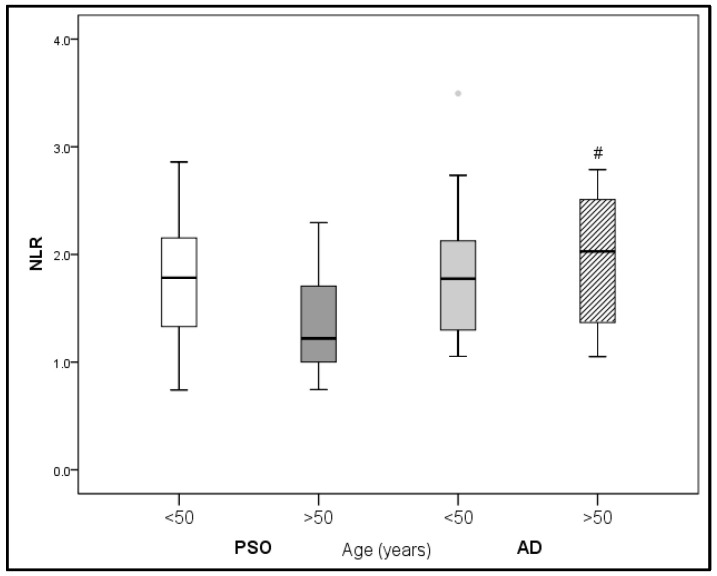
Comparison of NLR in PsO vs. AD according to age. ^#^ *p* < 0.05 vs. PsO (>50 years).

A comparison in NLR values between the PsO and AD subgroups according to age is presented in Figure 1.

Significantly higher NLR values were found in patients with AD older than 50 years in comparison with patients with PsO of similar age (*p* < 0.05, Figure 1).

**Figure 2:**
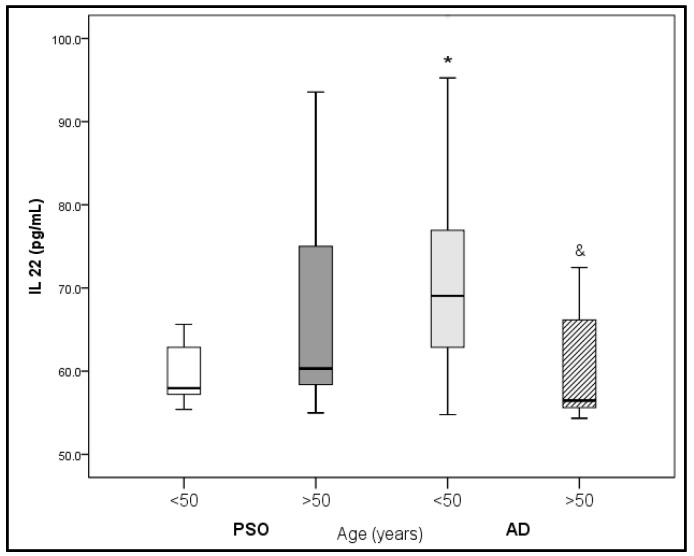
Comparison of IL-22 in PsO vs. AD according to age. * *p* < 0.05 vs. PsO (<50 years); ^&^ *p* < 0.05 vs. AD (<50 years).

Significantly lower IL-22 levels were found in patients with AD older than 50 years in comparison with patients with AD younger than 50 years. On the contrary, higher IL-22 levels were found in younger patients with AD (<50 years) compared with patients with PsO of similar age (<50 years) (*p* < 0.05, Figure 2).

Patients with PsO and with comorbidities had significantly lower PLT, PCT and PLR, whereas lymphocytes, MPR and RPR were significantly higher compared with PsO patients without comorbidities. Patients with AD and with comorbidities had significantly lower PCT and PLR, whereas RPR was significantly higher compared with AD patients without comorbidities (Table 3).

There was no difference in biomarkers of immunity and inflammation in the examined groups related to comorbidities (data not presented). Also, we did not observe a correlation between PASI score and any of the examined parameters (data not presented).

## 4. Discussion

As far as we are aware, ours is the first study to conduct a comparative analysis of hematological parameters and their indexes in patients with PsO and AD. Moreover, this is one of the rare studies that evaluated serum biomarkers of immunity and inflammation in patients with one of these two skin diseases.

We found no difference in the examined parameters between groups except for the mean corpuscular volume (MCV) which was higher in the PsO group vs. the AD group. Also, a higher neutrophil-to-lymphocyte ratio (NLR) was found in patients with AD > 50 years vs. patients with PsO of similar age.

Patients with PsO and with comorbidities had lower PLT, PCT and PLR, whereas lymphocytes, RPR and MPR were higher vs. PsO patients without comorbidities. Patients with AD and with comorbidities had lower PCT and PLR, whereas RPR was higher vs. AD patients without comorbidities.

Zhou et al. [8] found a positive correlation between the severity of PsO (i.e., PASI score), PLR and NLR, as well as the negative correlation with lymphocyte-to-monocyte ratio, respectively.

Neutrophils are involved in the immuno-inflammatory processes in the skin lesions of PsO, being a typical feature of this disease. They promote oxidative distress and favor pro-oxidants due to their ability to release reactive oxygen species [8]. Monocytes also contribute to the pro-inflammatory response by producing several cytokines (IL-6, TNF-α, IL-1, etc.) and monocyte-derived immune cells (i.e., macrophages and dendritic cells) that further secrete pro-inflammatory mediators (i.e., IL-23) and favor the onset of PsO [8].

Liu et al. [9] showed higher PLT, PCT and MPV in their PsO group in comparison with controls. They further confirmed a weak correlation between PASI and MPV, PLT and PDW, respectively. Higher PLT and lower NLR and PNR were also found in patients with AD [10].

Although the role of PLT in hemostasis is well-established, recent findings also support their involvement in regulation of immuno-inflammatory processes [3,4]. Lower PLT levels in PsO with comorbidities, as well as lower PCT and PLR in PsO and AD with comorbidities, could be explained by the accumulation of platelets at the sites of inflammation. Moreover, it was shown that pro-inflammatory cytokines (i.e., TNF-α) favor the activation of platelets, leading to the onset of thrombosis and cardiometabolic diseases [4,12].

Our results indicated higher RPR and MPR in PsO and higher MPR in both diseases with comorbidities compared with the patients without comorbidities. To the best of our knowledge, RPR and MPR have not been explored regarding PsO and AD so far. These two indexes of erythrocyte and platelet activation in a cardiometabolic setting could be increased in PsO and AD comorbidities as a consequence of prolonged impact of cardiometabolic-related inflammation [7]. In a recent meta-analysis, Yi et al. [13] found that RDW and MPV (i.e., indexes that are included in RPR and MPR, respectively) might be reliable predictive diagnostic parameters of PsO. However, similar to our findings, no relationship with PASI score was identified [13,14].

The association between RDW and the primary adverse cardiac events in patients with PsO was also confirmed and RDW was linked to the inflammatory status and clinical progression in patients with PsO [15]. In our recent study [7] we have shown that these novel markers of PLT reactivity and activation (i.e., RPR and MPR) are positively correlated with atherosclerotic lesion severity and complexity (i.e., as determined by Syntax Score).

We have not recorded a difference in inflammation (i.e., CRP) and immunity markers (i.e., IFN-γ and IL-22) between the PsO and AD groups in the current study.

Our results are in accordance with Krupka-Olek et al. [16] who did not find a difference in IFN-γ and IL-22 between PsO (*n* = 47) and AD (*n* = 45). Also, Bozek et al. [17] did not find a difference in IFN-γ between PsO (*n* = 28) and AD (*n* = 41) in a younger population, whereas children with AD exhibited more than twice as higher serum IL-22 levels compared with children with PsO, presuming that PsO underlies different pathological mechanisms from AD.

When we divided our patients according to age, we found higher IL-22 levels in patients with AD < 50 years vs. patients with PsO of similar age. Lower IL-22 levels were found in patients with AD > 50 years vs. patients with AD < 50 years. This finding supports the notion that the treatment for AD should be age-related. In line with this, Zhou et al. [18], in a large cross-sectional study that encompassed patients with AD (*n* = 5246) and healthy controls (*n* = 571), showed an age-specific decrease in IL-22 expression levels only in patients with AD, but not in the control group, whereas an increase in IFN-γ with age in both groups and positive correlation between IFN-γ and age was found. These findings are in accordance with some previous ones showing that innate immune cell-released cytokines are upregulated with age in individuals free of underlying systemic inflammation or infection [18,19,20].

Increased expression of IL-22 was also shown in younger patients with PsO in comparison with their older, adult counterparts [21].

Cytokine IL-22 was shown to be a potent stimulator of keratinocyte proliferation and IL-22-induced epidermal alterations similar to PsO were confirmed by animal studies [22,23]. The epidermal keratinocytes upregulation and proliferation leads to the induction of epidermis acanthosis via the activation of signal transducer and activator of transcription 3 (STAT3) in AD and PsO [24]. Moreover, the treatment with anti-IL-22 was found to be efficient in patients with AD, although it was not efficient in PsO [24,25]. There are assumptions that IL-22 is involved in pathogenesis of PsO and AD via different mechanisms [26]. Unlike Th17 cells that secrete both IL-22 and IL-17 in the skin of patients with PsO, Th22 cells in the skin of patients with AD enhance IL-22 expression followed by attenuated IL-17 expression leading to epidermal hyperplasia, a typical feature of AD.

Cytokine IFN-γ is regarded to be a major cytokine in PsO pathogenesis, being a bridge between inflammatory T-cells and keratinocytes and enabling the migration of T-cells into the epidermal lesions and altogether forming the primary plaque in PsO. During this pathological process, some T-cells and dendritic cells infiltrate epidermis, stimulate the release of pro-inflammatory cytokines and form psoriatic lesions [27].

We have found a positive correlation between IFN-γ and disease duration of PsO, but not with PASI score. Neither Kaur et al. [28] nor Kurtovic et al. [29] found a relationship between IFN-γ and PASI score, which is contrary to some other findings [30,31]. We did not find a correlation between IL-22 and PASI score either, unlike Wawrzycki et al. [32] who showed a positive correlation between IL-22 and PASI score. Additionally, some other studies showed a relationship between IL-22 and PASI score [33,34], as well as a decrease in IL-22 following treatment with methotrexate [35].

The discrepancy between the obtained results could in part be explained by different durations of each disease and associated comorbidities, sample size, variety in disease severity, differences in therapeutic treatment, etc.

The complexity of the individual profile of each patient with PsO or AD remains the major challenge when the treatment of these chronic skin diseases is concerned. The multifactorial pathophysiological nature of these inflammatory skin diseases, in addition to related comorbidities, emphasizes the importance of identifying those group of patients with enhanced inflammatory response. The quest for the biomarkers that would best reflect the severity of disease could provide better control of PsO and AD in such patients and enable adequate therapeutic approach and reduce the side effects and health costs [36,37].

Thus far, no consensus exists concerning the panel of biomarkers that could be used in routine everyday clinical practice. An algorithm that would include phenotype and genotype of each patient with PsO or AD could be the most reliable approach for each patient, using a personalized/precision medicine approach for such disorders [36,37].

Similar to many previous studies [16,17,28,29], the current study is limited for the relatively small sample size and for the causal mechanisms due to its cross-sectional nature. Additionally, we have attempted to overcome the age difference between the PsO and AD groups by further subdivision of the patients into patients younger and older than 50 years. We were not able to exclude other potential confounders, such as the effect of medications. Nevertheless, this is the first study that showed a higher pro-inflammatory state (i.e., higher NLR and IL-22) in AD vs. PsO in age-specific groups.

## 5. Conclusions

A higher pro-inflammatory state (i.e., higher NLR and IL-22) in AD vs. PsO in age-specific groups was shown. A higher pro-inflammatory state (i.e., as reflected by platelet indexes) was found in both diseases with comorbidities in comparison with those patients without comorbidities. The obtained results suggest that age-specific therapeutic treatment modalities could be beneficial in AD and PsO and that related comorbidities make an additional burden compromising a pro-coagulant state and increasing cardiometabolic risk. In light of these findings, platelet indexes could be reliable diagnostic biomarkers. Longitudinal large-scale studies are necessary to confirm such assumptions.

## Figures and Tables

**Table 1:** Hematological parameters in patients with psoriasis versus atopic dermatitis.

Parameter	PsO	AD	*p*
WBC ×10^9^/L	7.14 (5.51–9.25)	8.12 (5.41–9.13)	0.950
RBC ×10^12^/L	4.76 (4.38–5.07)	4.67 (4.40–4.99)	0.736
Hgb (g/L)	147 (129–154)	138 (125–147)	0.104
HCT (L/L)	0.444 (0.409–0.458)	0.424 (0.400–0.454)	0.229
MCV (fl)	92.1 (89.4–94.7)	90.6 (88.6–91.8)	0.040
MCH (pg)	30.0 (29.2–31.9)	29.4 (28.9–30.4)	0.064
MCHC (g/L)	329 (323–336)	323 (321–332)	0.129
RDW CV (%)	12.7 (12.3–13.6)	13.3 (12.6–13.7)	0.058
PLT ×10^9^/L	240 (200–308)	261 (213–313)	0.384
MPV (fl)	9.85 (8.95–10.65)	9.43 (8.85–10.15)	0.179
PCT (L/L)	0.0023 (0.0020–0.0029)	0.0024 (0.0022–0.0028)	0.503
PDW (fl)	11.1 (9.2–12.6)	10.4 (9.0–11.3)	0.173
Neutrophils ×10^9^/L	3.66 (2.60–4.89)	4.62 (2.52–5.07)	0.373
Lymphocytes ×10^9^/L	2.29 (2.04–2.98)	2.26 (1.85–2.56)	0.400
Monocytes ×10^9^/L	0.63 (0.51–0.79)	0.62 (0.51–0.76)	0.583
Eosinophils ×10^9^/L	0.19 (0.11–0.27)	0.19 (0.13–0.39)	0.335
Basophils ×10^9^/L	0.05 (0.03–0.07)	0.06 (0.03–0.06)	0.672
MPR	0.042 (0.032–0.051)	0.038 (0.030–0.045)	0.233
RPR	0.055 (0.043–0.079)	0.051 (0.043–0.063)	0.773

Data are presented as median (2575 percentile), *p*-value: Kruskal–Wallis test.

**Table 2:** Biomarkers of immunity and inflammation in patients with psoriasis versus atopic dermatitis.

Parameter	PsO	AD	*p*
CRP (mg/L)	1.55 (0.70–4.55)	1.80 (0.60–4.20)	0.682
NLR	1.51 (1.05–2.08)	1.77 (1.29–2.27)	0.100
PLR	100 (82–120)	109 (82–139)	0.252
IFN-γ (pg/mL)	20.3 (17.1–24.5)	19.0 (16.9–22.5)	0.381
IL-22 (pg/mL)	59.2 (57.6–69.0)	68.5 (57.0–76.5)	0.216

Data are presented as median (25–75 percentile), *p*-value: Kruskal–Wallis test.

**Table 3:** The influence of comorbidities on hematological parameters in patients with psoriasis versus atopic dermatitis.

Parameter	PsO		AD		*p*
	Without Comorbidities *n* = 19	With Comorbidities *n* = 21	Without Comorbidities *n* = 20	With Comorbidities *n* = 20	
PLT ×10^9^/L	265 (233–316)	223 (158–244) ^a^	267 (237–315)	234 (172–275)	0.059
PCT (L/L)	0.0027 (0.0022–0.0029)	0.0021 (0.0018–0.0025) ^a^	0.0026 (0.0024–0.0029)	0.0023 (0.0018–0.0024) ^c^	0.044
Lymphocytes (%)	31.1 (26.5–36.4)	37.4 (31.6–42.5) ^a^	29.9 (24.2–34.7)	33.5 (27.8–40.8)	0.030
PLR	112 (97–137)	96 (60–107) ^a^	124 (98–165)	96 (81–115) ^c^	0.008
MPR	0.033 (0.029–0.044)	0.044 (0.036–0.062) ^a^	0.038 (0.029–0.040)	0.040 (0.031–0.054)	0.052
RPR	0.050 (0.040–0.058)	0.057 (0.052–0.087) ^a^	0.050 (0.043–0.055)	0.058 (0.044–0.088) ^c^	0.103

Data are presented as median (25–75 percentile), *p*-value: Kruskal–Wallis test; ^a^ *p* < 0.05 vs. PsO without comorbidities; ^c^ *p* < 0.05 vs. AD without comorbidities (Mann–Whitney U test).

## Data Availability

Data are available upon reasonable request (contact person: aleksandranklisic@gmail.com).
